# Issues to Be Considered in Counting Burrows as a Measure of Atlantic Ghost Crab Populations, an Important Bioindicator of Sandy Beaches

**DOI:** 10.1371/journal.pone.0083792

**Published:** 2013-12-23

**Authors:** Maíra Pombo, Alexander Turra

**Affiliations:** Oceanographic Institute of the University of São Paulo, Department of Biological Oceanography, São Paulo, São Paulo, Brazil; James Cook University, Australia

## Abstract

The use of indirect estimates of ghost-crab populations to assess beach disturbance has several advantages, including non-destructiveness, ease and low cost, although this strategy may add some degree of noise to estimates of population parameters. Resolution of these shortcomings may allow wider use of these populations as an indicator of differences in quality among beaches. This study analyzed to what extent the number of crab burrows may diverge from the number of animals, considering beach morphology, burrow depth and signs of occupation as contributing factors or indicators of a higher or lower occupation rate. We estimated the occupation rate of crabs in burrows on nine low-use beaches, which were previously categorized as dissipative, intermediate or reflexive. Three random 2-m-wide transects were laid perpendicular to the shoreline, where burrows were counted and excavated to search for crabs. The depth and signs of recent activity around the burrows were also recorded. The occupation rate differed on the different beaches, but morphodynamics was not identified as a grouping factor. A considerable number of burrows that lacked signs of recent activity proved to be occupied, and the proportions of these burrows also differed among beaches. Virtually all burrows less than 10 cm deep were unoccupied; the occupation rate tended to increase gradually to a burrow depth of 20–35 cm. Other methods (water, smoke, and traps) were applied to measure the effectiveness of excavating as a method for burrow counts. Traps and excavation proved to be the best methods. These observations illustrate the possible degree of unreliability of comparisons of beaches based on indirect measures. Combining burrow depth assessment with surrounding signs of occupation proved to be a useful tool to minimize biases.

## Introduction

The attractiveness of sandy-beach environments and their particular geological traits make beaches highly susceptible to a wide range of local- to global-scale stressors, from tourism and vegetation removal to erosion and rising sea levels. Together with the limited number of marine reserves, the manifold stress factors make sandy beaches among the most threatened ecosystems in the world [1], [2]. According to Brown and McLachlan [1], improvements in the understanding and management of sandy-shore ecosystems may considerably minimize the effects of increasing human pressures. Defeo et al. [2] highlighted the prime importance of long-term monitoring to identify and quantify global-scale impacts on sandy beaches.

Assessing key groups of bioindicator organisms is an active area of sandy-beach studies. Some groups of supralittoral macrofauna, such as Talitridae, Cirolanidae and Ocypodidae [3] are particularly sensitive to impacts from tourism activities (trampling, beach cleaning), vehicle traffic, and shoreline armoring, erosion and nourishment [4]–[11]. Recent efforts of Research Networks, e.g. ReBentos (acronym for, in Portuguese, *Monitoring Network for Coastal Benthic Habitats*), also highlight the importance of improving and organizing knowledge of key organisms to aid in emerging management requirements [12].

The use of Atlantic ghost-crab populations as an indicator of beach quality and a tool for monitoring these environments has been widely proposed [13]–[18]. Ghost crabs are the most conspicuous invertebrates of sandy beaches in the tropics and subtropics, and an important top-down control factor [19]. They inhabit sandy beaches from the upper intertidal zone to dunes and vegetated areas, where they build burrows and maintain territories around them [20], [21]. The vast majority of burrows are Y-shaped, composed of a main branch and an alternative, closed one (rarely more), which may also open to the surface [22]. Reports on the main daily period of activity of these crabs state either that they are mostly active at night, or that no difference was detected over a 24-h cycle [21], [23].

The crabs' excellent camouflage, quickness and sensitivity to observers have necessitated wide use of indirect methods, which basically use the number and size of active burrows as the parameters for population assessments [13], [19], [20], [23], [24]. An active burrow is identified by the observation of typical traces left by the crabs, which include recently excavated sand and tracks surrounding the burrow, and also the definition of the perimeter of the burrow opening [20], [25]. The ease, non-destructiveness, and low cost of this censusing approach make these crabs even more attractive to study.

The Atlantic ghost crab *Ocypode quadrata* is the only member of the genus along the entire western Atlantic coast [21], so that interspecific competition, spatial overlapping and the difficulty of distinguishing between burrows of different species cannot bias population studies. Altogether, these crustaceans offer many advantages for estimating disturbances, and the great majority of recent research on *Ocypode* has focused on its use as a valuable impact-assessment tool.

However, certain shortcomings may impede comparisons among beaches or over time [26]. Although these are territorial animals, there is no definite information about the number of burrows that a single crab might actively maintain, for example, to increase the safety of its foraging area. Burrow counts might overestimate crab populations, and may also be subject to different ranges of errors in different locations.

There is evidence that population density varies according to beach type, but differently from other, smaller macrofaunal species, populations of *O. quadrata* do not change much in abundance according to the grain size of the sediment [3]. However, considering that grain size and soil compaction might significantly affect the effort needed to dig a burrow, and how long the burrow would last until it collapsed, beach morphodynamics could, in part, affect the proportion of burrows that are occupied. However, since this evidence is based essentially on burrow counts, it could partially reflect differential occupation rates resulting from natural environmental factors.

Not only external environmental factors, but some internal characteristics are interesting to consider along with the occupancy or not of a burrow. The signs of occupation surrounding burrows, i.e., fresh tracks, excavated sand and/or preserved perimeter definition, which are traditionally used to decide whether to count a burrow [20], [25], probably last for different periods of time in different beaches, being affected by grain size and slope. Sediment compactness, for example, may affect the number and depth of burrows.

Here, we attempted to assess to what extent the number of burrows might diverge from the number of animals (i) over different beaches, with different morphodynamics; and (ii) how some internal factors may indicate a higher or lower occupation rate, specifically (ii.1) signs of recent activity surrounding the burrow and (ii.2) burrow depth. Further, we (iii) attempted to measure the effectiveness of the excavation method used to estimate the occupation rate, by quantifying and comparing its results with several related methods. This study resulted in some simple but important observations on basic features that may be useful for further indirect studies of ghost-crab populations.

## Methodology

**Figure 1:**
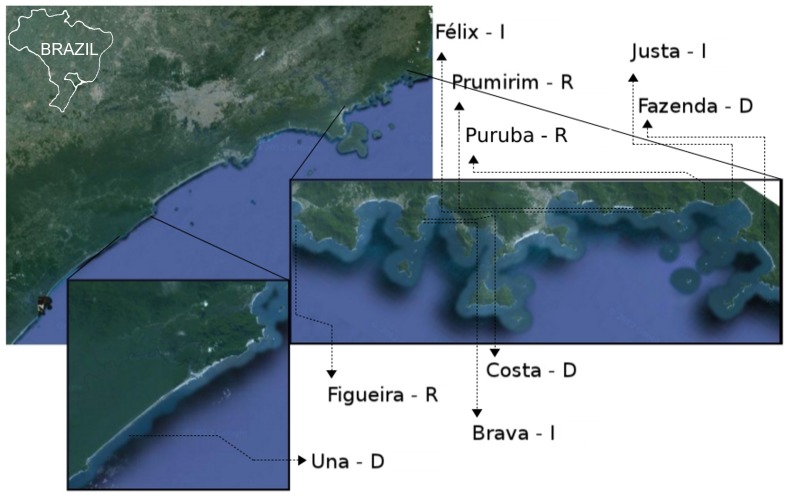
Study area. Satellite image of southeastern Brazil and detail of the study area. The nine beaches are between 24°31′S, 47°10′W and 23°22′S, 44°W50′. Each is named and followed by a letter indicating its morphodynamics: *D* – dissipative, *I* – intermediate and *R* – reflexive. Map data: Google, Cnes/Spot Image, DigitalGlobe, Landsat, TerraMetrics.

Nine low-use beaches were selected on the coast of São Paulo State, southeastern Brazil (Fig. 1). By studying preserved, low-use beaches, we attempted to focus on natural environmental factors rather than human impacts. The beaches (from 24°31′S, 47°10′W to 23°22′S, 44°W50′) were previously categorized as dissipative (Costa, Fazenda and Una), intermediate (Félix, Justa and Brava da Fortaleza) or reflexive (Figueira, Prumirim and Puruba). Fieldwork was conducted during daylight hours in late November and early December 2011, when the weather is typically warm with scattered rain showers. A homogeneous area 200 m long was selected on each beach, where three 2-m-wide transects were randomly laid out perpendicular to the shoreline, and every 2 m (in 4-m^2^ quadrats), burrows were counted, measured (depth, using a semiflexible steel cable), and excavated to search for the occupying crab. All burrows on a transect were counted, from the low intertidal zone to the vegetated area, until it was certain that no further burrows remained. All measurement activities and collections complied with the license from the appropriate federal environmental agency (*Ministério do Meio Ambiente (MMA) – Instituto Chico Mendes de Conservação da Biodiversidade (ICMBio*) No. 31629-1; acronyms for, in English: Ministry of the Environment – Chico Mendes Biodiversity Conservation Institute).

**Figure 2:**
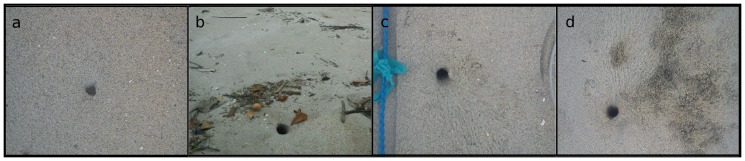
Categories of signs of occupation. Examples of each of the four categories of intensity of signs of occupation of *Ocypode quadrata*: a- none (no sign of occupation); b- subtle (barely recognizable tracks and/or excavated sand; only noting the definition of the internal perimeter); c- moderate (obvious presence of sand, or tracks, and internal perimeter definition); d- strong (presence of more than one clear sign and/or a very prominent one).

Since the study concerned a methodological approach, all burrows were assessed, including covered or semi-covered burrows. The signs of occupation around the burrow were recorded for Fazenda, Una, Brava, Justa and Figueira beaches, and later classified into four categories (Fig. 2): none – no sign of occupation; subtle – barely recognizable tracks and/or excavated sand; only noting the definition of the internal perimeter of the burrow; moderate – obvious presence of sand, or tracks, and a well-defined internal perimeter; strong – presence of more than one clear sign and/or a very prominent sign. Considering that the upper portion of a burrow is funnel-shaped, due to the unconsolidated sediment, which changes from beach to beach, we used “internal perimeter” for the perimeter of the lower circumference of the funnel, since from that point on the shape of the burrow best matches a perimeter modelled by the crab.

All burrows were excavated to the bottom whenever possible. The steel cable was helpful to locate the bottom of the burrow; probing around while excavating helped to detect an occasional continuation or side branch. Differences in soil compaction made it easier or harder to be sure that the crab was not there, but usually, it was easy to establish that the burrow was in fact empty, in the case of larger, shallower burrows. This led us to consider, in most cases where the crab was not found, two situations: (i) the burrow was empty in all cases – *minimum rate*; and (ii) the burrow was occupied in all cases - *maximum rate*. The difference between these values (minimum and maximum rate) represents an “uncertainty rate”.

For the purpose of evaluating the excavation method as a measure of occupation rate, three other methods, described below, were applied along with excavation, following the same transect pattern (three replicates of 2-m-wide transects comprised of 4-m^2^ quadrats). Samples were taken during three consecutive days, each comprising 4 transects, one of each treatment, in August 2012. The method giving the highest mean values of occupation and the lowest standard deviation was considered the most appropriate. The chosen area is a dissipative beach with restricted access (Arpoador Beach), within a Conservation Unit (EEJI - acronym for, in English: Juréia-Itatins Ecological Station), where tourist activity would not be a possible source of bias. These methods were:

- 1 – Flooding the burrow (water): a 5-L container was used to flood all identified burrows. Sign of occupation: crab left the burrow.
- 2 – Smudging (smoke): fumigator. Sign of occupation: crab left the burrow.
- 3 – Setting traps (trapping): a piece of cotton lint was used to close each burrow opening, which was also surrounded (by burying a thick cylindrical cardboard panel, around 60 cm in diameter). Traps were left in place for 24 h. Sign of occupation: the lint was disturbed.
- 4 – Excavating (excavate): The main duct was traced with the aid of a flexible steel cable. Moving the cable around while excavating enabled us to locate any alternative openings around the main duct. Sign of occupation: finding the crab.

All these methods are inexpensive and easily replicated, although trapping will increase in efficiency if a longer response time is allowed than for the other methods, and if the traps cannot be dismantled by visitors. Values were compared using one-way ANOVA, after assessing for its premises, and a Tukey post-hoc test.

Three other methods were applied, under different, more exploratory, conditions, attempting to credibly establish the crabs' presence or absence: the use of a bronchoscope, stethoscope, and ground-penetrating radar (GPR), with simpler experimental designs, to assess their feasibility and value. The bronchoscope and stethoscope were used in randomly chosen burrows and in burrows where a crab was known to be present. The GPR consisted of an armored antenna with 200 MHz frequency range and high resolution, and reached depths up to 6 m. Two sampling procedures were used: moving in straight lines across-shore, every 5 m in a 50-m beach section; and focusing on burrows where crabs were observed.

## Results

**Figure 3:**
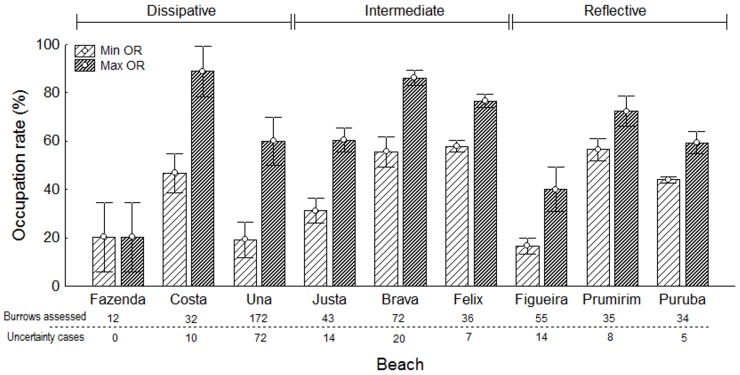
Minimum and maximum occupation rate by beach. Mean and standard error of occupation rate of *Ocypode quadrata* burrows (minimum and maximum rates), by beach. The total number of burrows assessed and the number of uncertainty cases, on each beach, are shown below the x axis.

The mean minimum occupation rate ranged from 20 to 60% for the study areas, and did not reach 40% in four areas (Fazenda, Una, Justa, Figueira). The mean maximum occupation rate showed a larger range over areas, from 20 to 90%, exceeding 80% in only two areas (Costa and Brava beaches). No pattern distinguishing rates by morphodynamics could be determined (Fig. 3), but the degree of uncertainty was somewhat greater for beaches with more dissipative profiles, except for Fazenda Beach, where the low abundance of individuals allowed exceptionally high accuracy.

**Figure 4:**
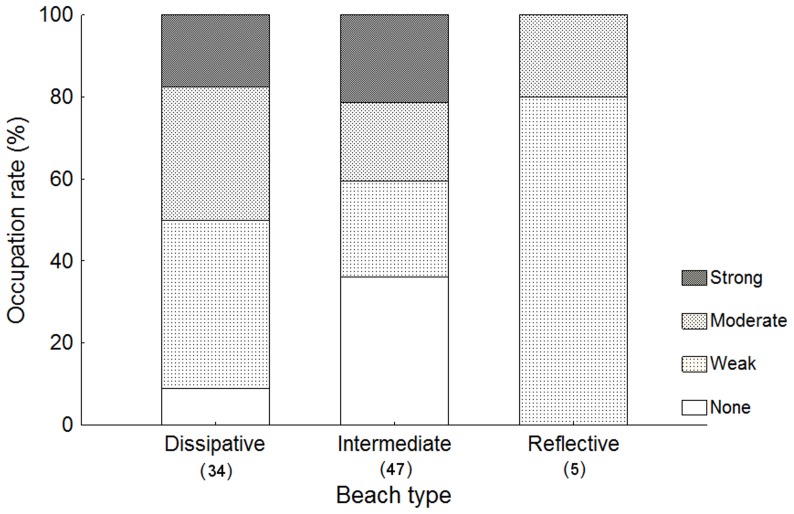
Relative intensity of signs around occupied burrows by beach type. Intensity of signs of *Ocypode quadrata* activity around occupied burrows. None – no sign of occupation; Subtle – only the internal perimeter definition and/or any other, barely recognizable sign could be noted; Moderate – Visible presence of sand and/or tracks; Strong – one of the above signs very obvious and/or presence of more than one visible sign. Covered or semi-covered burrows were also assessed. The number of animals caught on each type of beach is shown in parentheses below the x axis.

From the 274 burrows assessed for the intensity of signs of recent activity (excavated sand, fresh tracks, internal perimeter definition) 86 were proven to be occupied, of which 20 completely lacked any sign of activity. For the reflective beach, all occupied burrows showed at least subtle signs, although none showed strong signs (Fig. 4). However, combining all burrows without obvious signs of activity (no signs or subtle signs) revealed a tendency toward increasing occupation of these apparently inactive burrows, from dissipative to reflective beaches. Considering that subtle signs may be easily overlooked, intentionally or not, we observed that, for dissipative beaches, over 8.82% of burrows that lacked any signs of activity were in fact occupied, and this proportion reached 50% when burrows without obvious signs of activity were included. On intermediate beaches, this value increased from 36.17% to 59.57%, and on reflective beaches from 0% to 80%. On the reflective beach, few crabs were captured, but the data provided good evidence that a small proportion of the occupied burrows would show evident signs of activity in this beach.

**Figure 5:**
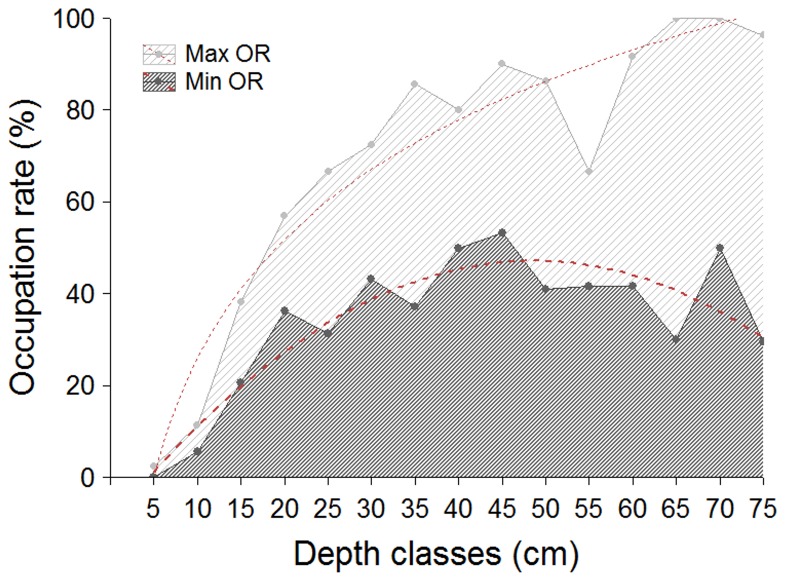
Minimum and maximum occupation rate according to depth. Occupation rates (minimum, dark gray; maximum, light gray) of burrows of *Ocypode quadrata*, according to their depth (n = 491); dashed red lines represent the curve trend of each rate with increasing depth.

Many burrows reached considerable depths, some over 1 m. The occupation rates increased gradually with burrow depth until 20 (minimum OR) or 40 cm (maximum OR) (Fig. 5), but virtually all burrows less than 10 cm deep were empty. With increasing depth the maximum rate tended toward 100%, and the minimum rate tended to decrease slightly.

**Figure 6:**
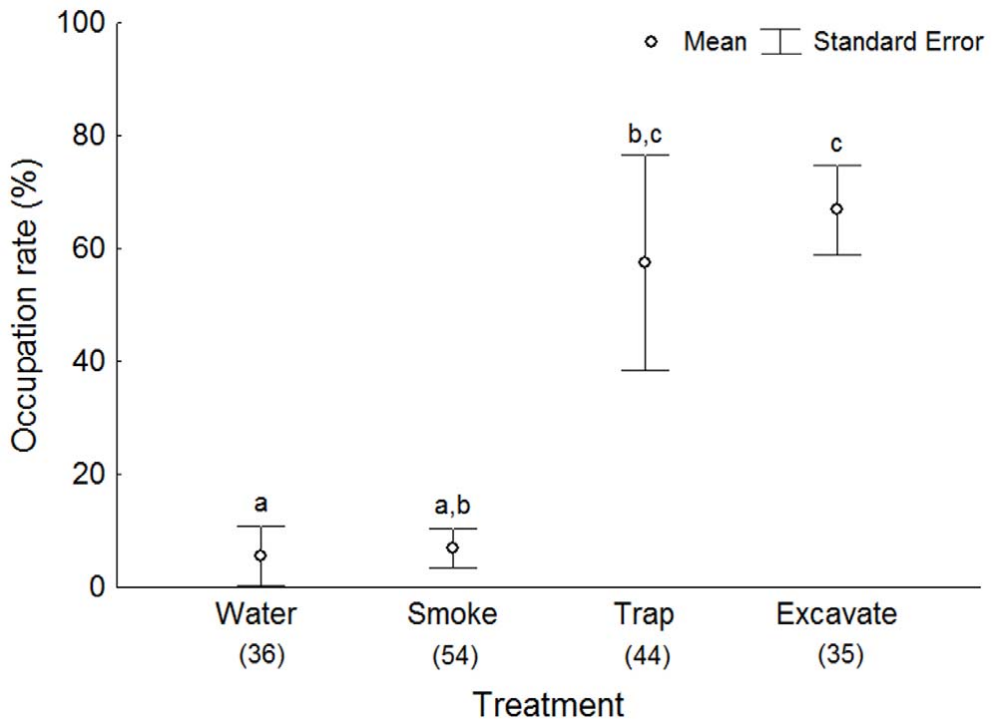
Occupation detected by means of different treatments. Mean and standard error of occupation rate of *Ocypode quadrata* burrows, determined by means of water, smoke, trapping and excavation methods, at Arpoador Beach in August 2012. Different letters denote significant differences discriminated by Tukey test. The number of burrows assessed for each treatment is shown in parentheses below the x axis.

A total of 169 burrows were assessed to compare methods for estimating the occupation rate. The homogeneity of variances (C = 0.78, d.f. = 3, p = 0.15) allowed the use of a one-way ANOVA, which indicated a significant difference among the methods (F = 8.17, d.f. = 3, p<0.01). A Tukey post-hoc test discriminated three similarity groups (Fig 6), of which flooding showed the worst result. The difference between smudging and trapping was marginal, and traps were significantly similar only to excavating (Table 1). Surprisingly, excavating showed the highest mean values and the lowest standard error. The bronchoscope, stethoscope and GPR did not give satisfactory results. The bronchoscope was used without the monitor, and therefore the light was not adequate and the image too small. Because the bronchoscope was about 60 cm long and some burrows exceeded 1 m, it was still necessary to excavate to some extent. The presence of branching tunnels may also impede the use of a bronchoscope, although this method may merit further attention. The stethoscope probably was not sensitive enough, and the ambient noise of water and wind are likely to interfere with the use of this tool regardless of its sensitivity. The GPR distinguished the air column in the burrows from the sand, rather than from the crabs inside. The use of different frequencies might improve the resolution of the objects, but would probably not change the actual results.

**Table 1:** Tukey post-hoc test between methods.

	Probabilities for Tukey post-hoc test		
Treatment	Smoke	Trap	Excavate
Water	0.9997	0.04815	0.0216
Smoke		0.0544	0.0243
Trap			0.9356

Approximate probabilities for Tukey post-hoc test to compare the occupation rate of *Ocypode quadrata* burrows by means of different treatments: water, smoke, trapping and excavation. Samples were taken at Arpoador Beach in August 2012.

## Discussion

Estimating the rates of occupied burrows may change the perspective on some parameters of *O. quadrata* populations over different beach types. The relationship between grain size and organism abundance and richness is well known; the supralittoral crustacean community is usually more abundant with increasing grain size. However, this pattern is not evident for *O. quadrata* [3]. Since ghost-crab population data are most often estimated by burrow counts [13], [19], [20], [23], [24], comparing abundance estimates among different beaches also involves the assumption that the relationship between burrows and crab density is similar among beaches.

According to the results obtained here, the occupation rate is indeed likely to differ among areas. Therefore, although differences in population density over different beaches may exist, they are likely to differ to some extent from those predicted through burrow counts. Better understanding of these differences can improve the usefulness of *O. quadrata* as a bioindicator or monitoring tool. Undoubtedly, the method of rate detection needs refinement, given the uncertainty of the excavation method, but no other methods, despite the additional investment in time (e.g., traps) or technology (e.g., GPR), were as effective as excavating to measure the occupation rate.

It seems reasonable that occupation rates may differ because of natural characteristics of the environment. On some beaches, or even in different zones of the same beach, burrows must collapse more rapidly than in others, especially when we consider that *O. quadrata* individuals exploit the entire supralittoral zone as well as dunes and vegetated coastal flats [20], [21], [26], [27]. Previous observations showed that an abandoned burrow can persist for over a week where the sediment is wetter or more compact, and especially if sheltered by vegetation (unpublished data). If this is so, the indirect method of assessing population density and age structure would be likely to produce higher estimates for some strata of the population than for others, since these crabs show size stratification across the beach [24], [26]. Therefore, abandoned burrows of animals of a length class that occupies drier regions would be prone to disappear faster than burrows constructed in wetter regions, and especially faster than those in vegetated areas, where the effects of wind and water are considerably reduced.

Combining these considerations, it seems reasonable that the minimum rate predicted by the present results is more reliable than the maximum rate. By this criterion, we have a scenario where at least 20% (this value is the same for the maximum OR), and probably not more than 60%, of the burrows of *O. quadrata* would be occupied.

An interesting, cautionary result emerged from the analysis of the intensity of signs of recent activity around burrows, since, in general, nearly a quarter of burrows lacking any sign were in fact occupied. Considering also subtle signs, i.e., those that are easily overlooked, this proportion tended to increase toward reflective environments, increasing from dissipative to intermediate beaches. On the reflective Figueira Beach, obvious signs of occupation were rare. This may reflect the ease with which the crabs leave tracks on finer-grained and more-compacted sediments. Also, it may be one more piece of evidence that burrows tend to close more easily on beaches with coarser sediment: if the signs disappear faster, the burrows should also close faster. If so, then dissipative beaches would tend to show lower rates of occupation of burrows of *O. quadrata* than reflective beaches, which partially agrees with the present results for minimum occupation rates.

**Figure 7:**
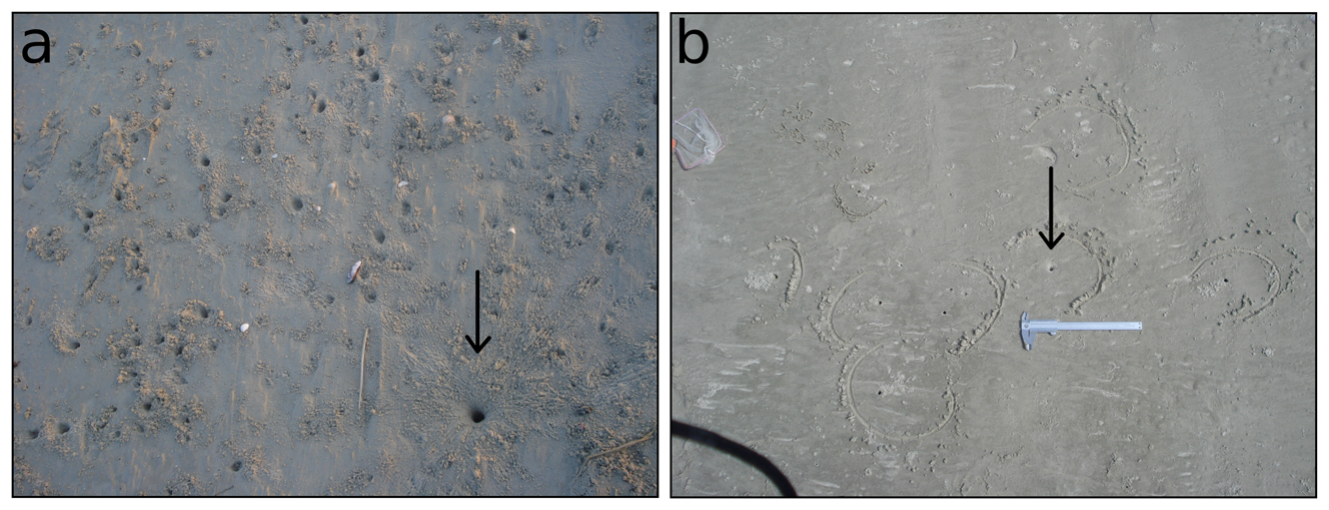
Examples of misleading situations. Examples of sets of burrows of *Ocypode quadrata* at Una Beach, southeastern Brazil. All these burrows showed signs of recent occupation, but only one was more than 10 cm deep and was the only occupied burrow (arrows). This may be more (a) or less (b) obvious from a superficial assessment.

The measurement of burrow depth showed a markedly different pattern for the maximum and minimum estimates of occupation rate. The differences between the curve trends may result from the increasing difficulty of reaching the crab; alternatively, the occupation rate of these deeper burrows may indeed be lower, since deeper burrows would last longer before collapsing. Furthermore, deeper burrows tend to be located farther from the waterline, where the sediment is less compact. Nevertheless, this measurement proved to be a helpful and easy tool to minimize biases arising from taking decisions of whether or not to consider a burrow based on signs of recent activity. Depth measurement helped, for example, in a location with high activity of crabs and a large number of burrows, to distinguish which burrows were started but abandoned from those that the crabs continued to dig (Fig. 7). Because virtually all burrows less than 10 cm deep were unoccupied, despite the intensity of signs around them, these burrows should not be counted in population assessments. Similarly, given the previous results, when no sign of activity is observed but the burrow depth is considerable, there is a greater chance that the burrow is occupied and therefore should be included in estimates.

Standardizing protocols that allow the use of ghost crabs as bioindicators is a challenge for researchers and managers of coastal environments. Although the results presented here illustrate the difficulties and noise in estimates and comparisons of crab density among beaches, stemming from differences in the occupation density, monitoring of ghost crabs through indirect methods may still be a powerful tool to evaluate temporal variations in crab densities in a given area, considering that the physical attributes (sediment, slope) of an area will not vary much over time. The results also show that noise can be reduced using simple biological features, such as depth, which should be associated with the traditionally used “signs of recent activity” in assessments of the Atlantic ghost crab.
